# Statistical Modelling of the Effects of Weather Factors on Malaria Occurrence in Abuja, Nigeria

**DOI:** 10.3390/ijerph17103474

**Published:** 2020-05-16

**Authors:** Oguntade Emmanuel Segun, Shamarina Shohaimi, Meenakshii Nallapan, Alaba Ajibola Lamidi-Sarumoh, Nader Salari

**Affiliations:** 1 Institute for Mathematical Research, Universiti Putra Malaysia, Serdang 43400, Selangor, Malaysia; oguntadeemmanuel2015@gmail.com; 2 Department of Statistics, University of Abuja, Abuja PMB 117, Nigeria; 3 Department of Biology, Faculty of Science, Universiti Putra Malaysia, Serdang 43400, Selangor, Malaysia; meenakshii@upm.edu.my (M.N.); lalabaajibolasarulam@gmail.com (A.A.L.-S.); 4 Department of Biostatistics, School of Public Health, Kermanshah University of Medical Sciences, Kermanshah 6715847141, Iran; n.salari@kums.ac.ir

**Keywords:** negative binomial models, weather variables, malaria, Nigeria

## Abstract

*Background*: despite the increase in malaria control and elimination efforts, weather patterns and ecological factors continue to serve as important drivers of malaria transmission dynamics. This study examined the statistical relationship between weather variables and malaria incidence in Abuja, Nigeria. *Methodology/Principal Findings*: monthly data on malaria incidence and weather variables were collected in Abuja from the year 2000 to 2013. The analysis of count outcomes was based on generalized linear models, while Pearson correlation analysis was undertaken at the bivariate level. The results showed more malaria incidence in the months with the highest rainfall recorded (June–August). Based on the negative binomial model, every unit increase in humidity corresponds to about 1.010 (95% confidence interval (CI), 1.005–1.015) times increase in malaria cases while the odds of having malaria decreases by 5.8% for every extra unit increase in temperature: 0.942 (95% CI, 0.928–0.956). At lag 1 month, there was a significant positive effect of rainfall on malaria incidence while at lag 4, temperature and humidity had significant influences. *Conclusions:* malaria remains a widespread infectious disease among the local subjects in the study area. Relative humidity was identified as one of the factors that influence a malaria epidemic at lag 0 while the biggest significant influence of temperature was observed at lag 4. Therefore, emphasis should be given to vector control activities and to create public health awareness on the proper usage of intervention measures such as indoor residual sprays to reduce the epidemic especially during peak periods with suitable weather conditions.

## 1. Introduction

Malaria is a life-threatening infectious disease caused by a single cell parasite and transmitted by bites of female *Anopheles’* mosquitoes from infected individuals to non-infected persons. The dynamism of transmission of malaria is an open problem. Studies have attributed the stable transmission in Sub-Saharan Africa (SSA) to changes in weather parameters while others identified non-weather-related factors [1]. The spatial variability of the weather parameters with time opens it for further consideration. Hence, the study of the influence of climate variability on the outbreak, resurgence and transmission of malaria is of paramount importance.

In 2017 there were about 219 million malaria cases worldwide which resulted in 435,000 deaths. About 90% of the global deaths occurred in SSA, with Nigeria having a considerable percentage of the global cases [2,3]. Global interventions, control measures and initiatives in recent times have led to a significant reduction in disease cases. Children aged less than five years, pregnant women, people with other ailments, travellers from non-endemic nations, refugees and displaced persons are the most susceptible to malaria [4].

Malaria is a common infectious disease, especially in the tropics where the prevailing environmental conditions are favourable for disease transmission. The impact of global warming and constant changes in weather conditions enhance the breeding, survival and spread of the pathogenic parasites [5]. Also, resistance of the host vectors to insecticides and resistance of the stubborn species of *Plasmodium* to anti-malarial drugs foster the current prevalence of the epidemic in the developing world especially in SSA nations where the disease trend constitutes health problems [6,7,8]. The disease persists as the prevalence of malaria infection; disease incidence and mortality from severe malaria cases are still very high in endemic nations [9]. Despite control interventions instituted to curb transmission of the pandemic, and its socio-economic impact globally, the region of SSA is facing challenges in reducing the intensity of the disease especially in remote areas where there is a lack of good understanding of the disease epidemiology, a weak health system, scarcity of control intervention measures and high poverty level amongst other factors [10].

There are various control strategies in place in SSA nations to curb the menace of the disease. Such interventions amongst others include vigorous health awareness, free distribution of insecticide-treated nets, indoor residual spray (insecticides), case management with artemisinin-based combination therapy, regular treatment and availability of intermittent preventive therapy in pregnancy [11]. Despite the control measures in place, high poverty levels, low levels of education and health awareness, negative attitudes, cultural practices at the community level, religious beliefs and political instability constitute a significant hindrance to malaria control in SSA [12]. Likewise, the resistance of vectors to insecticides and parasites to drug and spatial variability of weather parameters also hinder vector elimination campaigns in SSA [6,13].

The world has witnessed changes in climate factors in recent times with Africa and other tropical parts of the globe severely hit by its negative consequences on human existence and health [14,15]. The cumulative effects on humans are substantial as it affects the entire ecosystem [16]. For example, certain disease outbreaks such as the recent Zika virus disease, dengue fever and the resurgence of epidemics such as pneumonia, malaria and rift valley fever are associated with changes in weather variables [17]. The potential influence of change in weather parameters includes drought, increase in temperature, massive extinction of certain biological organisms and reduction in human longevity through direct exposure to excessive rays from the sun. Others are hunger and starvation as agricultural activities are adversely affected, increase breeding of the ectothermic definite host vectors like ticks, flies and mosquitoes as survival and reproduction rates of such arthropod species hang on the habitat suitability, abundance and biological distributions in space and time [5,18]. Weather variability also affects pathogens like *Plasmodium*, dengue fever viruses. Therefore, this aids the transmission of vector-borne infectious diseases likes malaria, Zika virus disease and dengue fever. The pathogens’ developmental processes, survivals and reproduction within the vector are enhanced at certain climatic thresholds [19,20]. For example, the average temperature between 18–30 °C favours breeding of malaria parasites [21,22]. Also, relative humidity of 60–90% can enhance the breeding and multiplication of *Plasmodium* parasite while a relative humidity below 60% decreases their population [6,23].

Early developmental stages of mosquitoes depend mainly on water availability, while the adults’ survival relies on temperature and relative humidity [11,20]. Rainfall can sometimes inhibit the survival and distribution of mosquitoes, especially when a saturating point is reached. The rainfall saturating effect can cause excess rainfall to reduce the number of mosquitoes by flushing away eggs and larvae in breeding sites and thus interrupt their developmental stages [6,18,24]. Also, ozone depletion and influences from El Niño cycles affect atmospheric air temperature, which in turn impact the epidemics of diseases.

Several studies are available in the literature on the complex interrelationship between malaria occurrence and various weather variables [15,25]. For example, Gunda et al. [26] appraised malaria incidence, its trends and association with weather variables in three rural districts in SSA. The study found a significant association of reported malaria cases with weather parameters. The malaria incidence was shown to be correlated with precipitation and mean temperature at some specific lag periods. Likewise, Akinbobola and Omotosho [6] examined and compared the relationship between weather variables and reported malaria cases at two different stations, each from different geopolitical zones in Nigeria. The results based on time-series analysis revealed a significant increase in malaria cases due to the potential influence of changes in weather variables. The study identified rainfall and humidity having positive association in both stations while maximum temperature had both inverse and direct relationships in the south and the north stations, respectively. Also, a recent study in Uganda revealed strong associations between malaria incidence and climate variables [6]. The authors employed both Poisson and negative binomial regression to investigate the potential relationship between the response and weather predictors. The study found a strong positive correlation between malaria cases and weather variables. A unit increase in both rainfall and average temperature leads to an increase in disease cases.

The presence of malaria is highly influenced by the anomalies of rainfall, temperature and humidity in SSA where Nigeria is situated [6,27]. These irregularities have aided the adaptation and prevalence of the malaria vectors and the parasite [28], thereby producing a resultant effect on disease epidemics. Previous studies in Nigeria reported variations in rainfall patterns to be above average in both the Central and Southern Nigeria while rainfall below average was reported in the Northern part of Nigeria [29]. A tropical wet and dry climate in Abuja and its environment provide a suitable environmental condition for malaria transmission which yields seasonal variation in the rainfall patterns within the non-arid region which in turn produce inter-annual (temporal) variability in epidemics [11,28,30]. The structure of the climatic factors gives support to the rate of survival of the vectors and the parasites. Malaria is endemic in Nigeria with the majority of the confirmed cases (over 90%) caused by *P. falciparum*, and the primary vectors are *Anopheles gambiae, Anopheles arabiensis* and *Anopheles funestus* [31,32].

Similarly, an increase in rainy days and daily temperature in Central Nigeria was significantly associated with an increase in *P. falciparum* malaria in Abuja [33]. Evans and Adenomon [25] reported an increasing trend of about 6% in malaria incidence in Central Nigeria during the rainy season while Nanvyat et al. [30] reported cyclic trends in malaria cases and its lagged associations with weather variables in Jos, Central Nigeria. Nmadu et al. [28] also reported a high prevalence of malaria parasitaemia among children during the peak of the rainy season (June and July) in Abuja. Furthermore, Nmadu et al. showed that *P. falciparum* is predominant among the children who were tested, 62.5% tested positive to *P. falciparum* while only 1.5% tested positive to *P. malariae*, and all other species of *Plasmodium* were absent.

The spatial variability of weather variables and steady changes in weather parameters necessitated the need for regular examination of such variables related to vector-borne diseases to enhance malaria early-warning systems and provide information on the changing malaria situation [26]. The subject matter has been studied extensively in the literature [1], nevertheless there exist uncertainties regarding the disease trend in the near future. This study is useful with a view to appraising known intervention put in place by health service providers and also helping policymakers design an appropriate intervention programme on vector control and proper case management. Therefore, the study examined the relationship between monthly malaria incidence and weather variables.

## 2. Materials and Methods

### 2.1. Study Area

The study location for this study is Abuja. Abuja is the federal capital territory of Nigeria and falls between the latitudes 9°20′ N and 7°25′ N of the equator and longitudes 5°45′ E and 7°′ E of Greenwich Meridian. It has a landmass of approximately 7315 km^2^ [33]. The official population was 776,298 people at the 2006 population and housing census [33,34]. The vegetation of Abuja is guinea savannah with limited forest areas [35]. The climate of the study area is tropical: non-arid climate with only two seasons throughout the year: wet and dry. The mean temperature in the study area ranges between 30–37 °C yearly with the highest temperature in March and the mean total annual rainfall of approximately 1650mm per annum [36].

### 2.2. Data Management

The data on monthly malaria incidence in Abuja for the period of January 2000 to December 2013 were collected from the diagnostic unit of the National Institute for Pharmaceutical Research and Development (NIPRD) Abuja, Nigeria. The malaria data obtained from NIPRD consist mainly of the confirmed monthly cases of *P. falciparum* with a few cases of *P. malariae*, which supports the discovery of prevalent malaria parasite in Abuja [28,33,37]. The diagnostic procedures were based on the laboratory analysis of blood samples of patients screened for the malaria parasite by examining the patient’s blood, spread out as blood smear under the microscope, which is the most common and accurate diagnostic test for malaria [28]. The weather data were also obtained for the same period from the relevant government agency in Abuja. Data on monthly rainfall (mm), temperature (°C) and relative humidity (%) were collected from federal capital territory Agricultural Development Project Gwagwalada, Abuja. The response variable used for the trend analysis via generalized linear model is the monthly incidence of malaria while the explanatory variables are rainfall (mm), temperature (°C), and relative humidity (%).

### 2.3. Statistical Analysis

The Poisson and negative binomial models are generalized linear models appropriate for modelling trend when rare discrete events for a specified time length are involved, such as malaria incidence data. For this study, the malaria counts were randomly observed for each of the 168 months; malaria incidence on a particular month was considered as independent of the next subsequent months. Based on these distribution assumptions, the Poisson and the negative binomial models were appropriate statistical tools for the analysis of the incidence data. For the Poisson model (PM), the response variable assumed a Poisson distribution and used logarithms as a link function for its mean value.

Poisson has a unique property of equal value for both the mean and the variance and assumes large counts to be a rare event [25]. These strong PM assumptions, however, relate to observations based on experimental and field studies and are always skewed (variance greater than mean) in the distribution of their observations with a long-tail. This may be due to unobserved heterogeneity or count data models [38]. The overdispersion with long-tail issue renders PM an inappropriate tool as it causes estimation error of the parameters. To remedy these issues, this study explored a more flexible tool for the modelling of the variance than PM. The negative binomial model (NBM) has an extra parameter (shape parameter that modifies variance independently from the mean) than PM. Thus, NBM accounts for overdispersion and long-tail distribution in the data. This makes NBM more robust than PM in modelling count outcomes with overdispersion problems.

The Poisson generalized regression model is given as:

(1) $$\vartheta =E\left(m|z_{1},z_{2,}\ldots ,z_{p}\right)=e^{Z^{1}\beta }$$

(2) $$ln\vartheta =Z^{1}\beta$$

where ϑ is the conditional mean of Poisson regression model, β is column vectors of regression parameter given Z, a vector of independent variables and m is the count response with only non-negative integer values. Maximizing the log of likelihood function gives estimates of the parameters of the model with the maximum likelihood estimation method [39].

Using the Pearson correlation coefficient, the relationships between the malaria incidence and weather variables based on each month of the year with no month lag were examined.

The pre-whitening of the data from the weather covariates was carried out to select all the significant lags of the predictors to be considered as covariates for model construction based on the values of the cross-correlation function of the filtered series. Autoregressive integrated moving average (ARIMA) models were fitted to each of the predictors to pre-whiten them and reduce their residuals to white noise [40], thereby, filtering out the effects of serial autocorrelation structure on the assessment of statistical significance. 

Following pre-whitening, cross-correlation analysis was performed at different time lags for the malaria incidence and weather variable in SPSS version 22. The significant lags were identified and included in the model building. The order of the ARIMA model was chosen by minimizing the Akaike information criterion (AIC). 

For the model fit and diagnostic, the residuals and their dependence were examined with time series plots. The residual analysis was performed with autocorrelation function (ACF) and partial autocorrelation function (PACF) of the residuals (adjusted). A good model yields non-correlated residuals with properties that resemble a white noise process. Likewise, the Ljung–Box test for residuals was also used to check if the model is correctly specified at 5% level of significance. A significant value indicates the presence of a structure in the residuals (adjusted) that the model failed to account for; therefore, the model did not adequately fit the data.

### 2.4. Ethical Clearance

This research relies exclusively on information readily available to the public. The data used were exclusively information about anonymous human subjects which does not require ethical review and clearance.

## 3. Results

### 3.1. Descriptive Statistics

**Figure 1:**
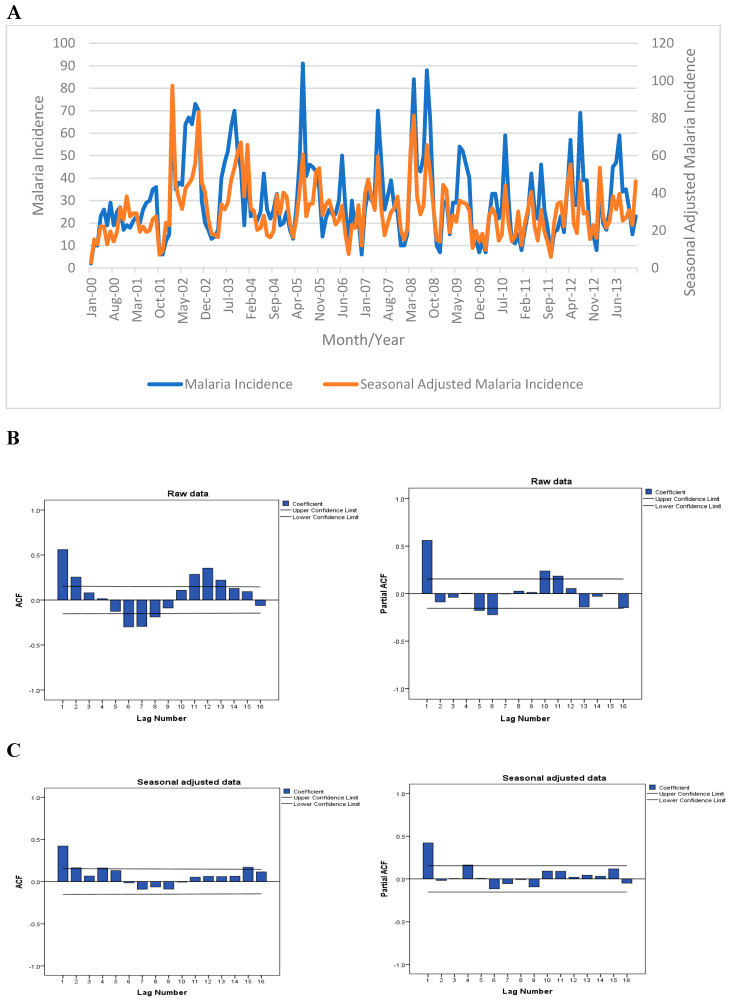
Malaria cases reported in the study area (2000–2013). (**A**) Malaria incidence: original and seasonally adjusted data (2000–2013); (**B**) Autocorrelation and partial autocorrelation functions computed from the raw data (cases); (**C)** Autocorrelation and partial autocorrelation functions computed from the seasonally adjusted data.

**Figure 2:**
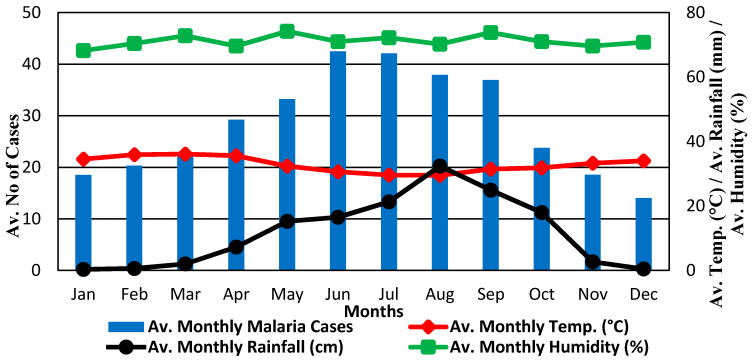
Relationship between the monthly average of malaria incidence and weather variables.

From January 2000 to December 2013, there were 5130 malaria cases reported in NIPRD, Abuja, with the monthly average of about 31 cases (Figure 1). The malaria data collected revealed the two seasons of the year in Nigeria: the wet and the dry seasons (Figure 2). More malaria cases were recorded in the wet season than the dry season. Also, the highest values of rainfall and humidity were recorded in the wet season while the reverse was true for temperature (Figure 2). The various data used for this study were time-series data, as such, fluctuations and seasonal patterns were observed in the original data. For the monthly incidence, a seasonal malaria incidence was observed with spikes and lower troughs at the peak of the wet and dry seasons, respectively. A sideways trend was evident in the malaria incidence between 2000 and 2013 with marginally increasing trend line (Figure 1). Based on these data, it was imperative to adjust for the seasonal and random variations in order to reveal the underlying trends and cycles. The irregular variations and seasonal effects in the observations were adjusted and the seasonal index was computed to remove the seasonal and other unwanted components. 

### 3.2. Model Outcomes and Information

The descriptive statistics for the response variable revealed a violation of the model assumption for the PM. The estimated values for the mean and the variance differed significantly. The result based on deviance statistics showed inappropriateness of PM for the trend analysis. A robust generalized regression method for modelling count data with some set of explanatory variables was used to handle the over-dispersion problem. The additional parameter in the negative binomial regression handles such issues and gives NBM an advantage over the usual PM.

The likelihood ratio test revealed the overall fit of the model (-2LL = 308.05, *p* < 0.001). All the explanatory variables were statistically significant (*p* < 0.05) (Table 1).

(3) $$\log_{e}Y_{\mathrm{Cases}}=4.603+{0.001X}_{\mathrm{Rainfall}\;}-{0.060X}_{\mathrm{Temperature}\;}+{0.010X}_{\mathrm{Humidity}}$$

The parameter estimates for the NBM is presented in Table 1. The results revealed an increase in malaria incidence of about 1.001(95% CI, 1.000–1.001) times for a unit increase in rainfall per month. Likewise, the disease cases increase significantly for an extra unit increase in relative humidity per month (1.010 (95%CI, 1.005–1.015), *p* < 0.001). On the other hand, there was a decrease in odds of malaria incidence of about 5.8% for a unit rise in temperature. 

**Figure 3:**
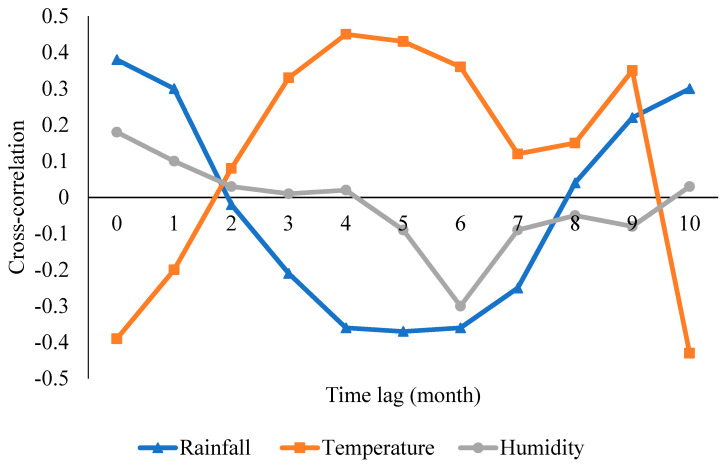
A Cross-correlation coefficient of malaria incidence and weather variables at different time lags (months).

Based on series pre-whitening, cross-correlations were used to examine the lagged associations between the weather variables and malaria cases at different lags (pre-whitened series) (Figure 3). The significant lags (1, 4 and 5) were identified and considered in the model building. Temperature with 4-month lag has the maximum positive cross-correlation value, while rainfall at lag 1 has the highest positive correlation with malaria incidence. At lag 5, there was a negative cross-correlation value between rainfall and malaria incidence, and this relation has the highest significant decrease in malaria cases with rainfall.

Following the results of the pre-whitening of malaria incidence and weather variables, the influence of weather variables on malaria incidence was also examined at different month lags. In Figure 3, only lags 1, 4 and 5 are significant lags. Thereby, only these lags were included as the covariates (Table 2). At lag one month, only rainfall significantly increased the incidence while both rainfall and temperature had a significant influence on malaria incidence at lags 4 and 5. The strongest positive weather influence was from the temperature at lag 4. Relative humidity significantly increased the cases at lag 4 while there was a negative influence of rainfall on malaria incidence at lags 4 and 5. The effect of both rainfall and humidity on malaria incidence decreased marginally with increasing lag months (Table 2). 

## 4. Discussion

The findings of this current study revealed the interrelationships of meteorological variables with malaria occurrence in Abuja. The results also identified key weather predictors of the malaria epidemic in Abuja for the two seasons of the year. The findings revealed a higher monthly incidence in the wet than in the dry season. This result is consistent with previous results in a similar study in Abuja [33], Ethiopia [41] and North-West India [42]. This current finding could be as a result of the adequate availability of water in ponds and ditches, canals and gutters, green vegetation around homes, places of work and construction sites. Therefore, these conditions increase the breeding of mosquitoes and disease transmission. Likewise, it could be because the wet season coincides with the planting season for local farmers in Abuja; as such, farmers were more vulnerability to malaria infection as the level of exposure to mosquito bites increases during these periods. The regression results based on NBM revealed positive relationships of rainfall and relative humidity with malaria incidence in Abuja, while every unit increase in monthly temperature reduces the incidence by about 5.8% monthly. This could be explained based on the biological ground, the breeding of the vectors is inhibited if the temperature rises above a certain threshold and it lowers the disease transmission at that point in time [9,11]. Temperature above 24 °C currently experienced in the study region, especially in the dry season, may be responsible for the observed trend in this study. A higher temperature has been shown to inhibit strongly the developmental stages of the mosquito parasite and its vectors (predominantly *Anopheles gambiae* and *Anopheles arabiensis*) across Nigeria [43]. For instance, a temperature above 30 °C shortened sporogonic stage of the *Plasmodium* parasite while the survival of mosquitoes is adversely affected, and its life cycles are not completed [6]. This finding of the current study is in agreement with previous related studies in Nigeria [21] and New Zealand [44]. The study reported a decrease in malaria incidence of about 4% for every unit rise in temperature in Nigeria.

Relative humidity is one of the major determinants of malaria incidence among the weather factors examined at zero lag. Based on the data from the study area; relative humidity is between 60% and 86% which is the most suitable threshold for the *Anopheles* mosquitoes to thrive and for the *Plasmodium* parasite to transmit the infection. Thus, relative humidity has the highest number of positive correlations with malaria incidence for all the months of the year, with exceptions in March and October. This is similar to the result observed in Nigeria [6]. The authors identified relative humidity amidst other potential malaria predictors of having the most significant influence on the survival of the host vector. However, this result is inconsistent with an earlier study in Ondo State, Southwest Nigeria [11]. The authors found wind speed as the most influencing predictor of malaria.

From the results of this study, malaria incidence in April to July is positively correlated with temperature. This agrees with a recent study in Nigeria [6]. However, the existence of an indirect relationship of the response with temperature in the remaining months in the rainy season (August to October) disagrees with a related study in Lagos, Nigeria [45]. The plausible explanation for this may likely be the confounding effects of the third factor ‘rainfall’ in the wet season. The saturating effect of rainfall in these months causes a decrease in incidence even in the presence of a suitable temperature as vectors are adversely affected. This is in agreement with the previous results documented in the literature [18,24]. Likewise, the correlation results of monthly malaria incidence and rainfall agree with the results previously documented in Abuja [33]. The high incidence recorded in the wet season and the low malaria incidence obtained in the dry season could be attributed to seasonality effects. Also, prolonged drought and excessive rainfall may inhibit the developmental stages of mosquitoes and eventually reduce the disease cases.

The initial results revealed that the malaria cases were observed throughout the year (during both the dry and the wet seasons) with varying degrees of incidence in the study area. The prevailing weather conditions mainly influenced these incidences. However, there was a reversed shift between the malaria incidence series and rainfall series, especially at the peak of the rainy season (August) and the months preceding it. This could be explained based on the fact that the excess downpour (rainfall) in this month may likely wash away the breeding sites of the primary vector. Hence, this may affect the number of cases recorded in this particular month of the year as an increase in rainfall may fail to produce additional cases of malaria. This is consistent with a related study in Nigeria [6]. Likewise, the plausible reason for the average number of cases observed in June (peak malaria month) may be the resultant effects of rainfall and other weather conditions in the preceding months. This agrees with a related study in Abuja [33]. 

The significant association between malaria incidence with 1-month lagged rainfall in this study is consistent with a related study in Zimbabwe [26]. This 1-month lag effect of rainfall could create enough time frames required for the developmental stages of the local vector mosquito (mainly *Anopheles gambiae* and *Anopheles arabiensis*) and the completion of the life cycles of *P. falciparum* within the vector. At 4-month lag, the temperature was significantly associated with malaria incidence. This is similar to the longer lagged effect of temperature on malaria transmission reported in Plateau State, Nigeria [30]. This could be explained based on biological grounds as a period of increased temperature, humidity and rainfall at defined intervals are usually followed by a period of a malaria epidemic. These high malaria risk periods are products of the resultant effects of the interactions between the weather parameters, vector mosquito and malaria parasite in the previous months.

There are different species of *Anopheles* mosquitoes discovered and reported in Nigeria [32]. However, *Anopheles gambiae and Anopheles arabiensis* remain the two main malaria vectors and the most important *Anopheles* species in malaria transmission across Nigeria [43,46].

The essence of early warning of malaria is to create a new mitigation mechanism to reduce the vulnerability of people at the risks posed by a malaria epidemic in the study region. Therefore, the current study provides valuable early warning information to health service providers and the general public on the likelihood of an occasional outbreak of malaria. Necessary precautionary measures become imperative due to the significant association of malaria cases with weather parameters.

The results of this study have to be handled with caution as it has some shortcomings. First, the analysis used secondary data which may likely be prone to some errors. Systematic and other errors due to the reliability of the laboratory procedures can sometimes be associated with an experimental error. Similarly, bias in experimental measurements or readings in the laboratory can occur. Also, unknown to users, secondary data can be presented in a summarized or modified version of the raw data. However, secondary data, if well measured and appropriately presented, can be precise, accurate, unbiased, reliable, valid and timely. Also, the data were only available on a monthly basis which may not reveal the actual variations embedded in the various observations. Other potential risk factors of malaria were not included in the regression model with weather variables for a holistic assessment of critical drivers in Abuja. Factors such as demographic characteristics, socioeconomic variables and public health control measures, immigration–emigration, changes in housing, land use, topography and subjects’ knowledge, attitude and practice regarding malaria may have influenced the response in terms of interest or associated with weather variables. However, this study provides baseline health information for future malaria intervention planning in Abuja.

## 5. Conclusions

The study revealed the link between malaria incidence and climatic predictors at different lags in Abuja. Relative humidity was identified as the primary driver of the malaria epidemic at lag 0 while the most substantial significant influence of temperature was observed at a 4-month lag. Also, the influence of rainfall and humidity on malaria epidemics decreased with lag months. Emphasis should be given to vector control mechanisms and public health awareness on proper usage of intervention measures to reduce the seasonal malaria incidence stimulated by suitable weather conditions.

## Figures and Tables

**Table 1:** Negative binomial regression results.

Model Predictor	Model Coef (β)	Std Error	Wald Chi-Square	Exp. (β)	95% CI	*p*-Value
Intercept	4.603	0.321	206.117	99.821	53.247,187.13	<0.001
Rainfall	0.001	0.000	25.945	1.001	1.000,1.001	<0.001
Temperature	−0.060	0.007	63.243	0.942	0.928,0.956	<0.001
Humidity	0.010	0.003	14.078	1.010	1.005,1.015	<0.001

**Table 2:** The influence of weather variables on malaria incidence with lagged-months in Abuja (2000–2013).

	Lag 1		Lag 4		Lag 5	
Variables	IRR (0.95CI)	*p*-Value	IRR (0.95CI)	*p*-Value	IRR (0.95CI)	*p*-Value
Rainfall	1.001 (1.001–1.001)	<0.001 *	0.999 (0.999–1.000)	<0.001 *	0.999 (0.999–0.999)	<0.001 *
Temperature	0.988 (1.974–1.002)	0.105	1.079 (1.064–1.095)	<0.001 *	1.065 (1.050–1.080)	<0.001 *
Humidity	1.005 (1.000–1.005)	0.068	1.006 (1.001–1.011)	0.016 *	0.998 (0.993–1.004)	0.556

IRR: Incidence rate ratio, CI: Confidence interval, * Statistically significant at *p* < 0.05.
